# Scleroderma calcinosis cutis score (SC^2^S): an imaging metric to quantify SSc-calcinosis cutis

**DOI:** 10.1093/rheumatology/keag302

**Published:** 2026-06-11

**Authors:** Ian D Odell, Crystal Cheung, Megan Wu, Stephanie Perez, Agrani Dixit, Cassandra van Horn, Muhammad Hamdan, Ilayda Gunes, Sophia Kujawski, Hyojeong Lee, Annie Wang, Denise Esserman, Michael Zamani, Francis Perry Wilson, John Onofrey, Xenophon Papademetris, Monique Hinchcliff

**Affiliations:** Department of Dermatology, Yale School of Medicine, New Haven, CT, USA; Department of Internal Medicine, Section of Rheumatology, Allergy & Immunology, Yale School of Medicine, New Haven, CT, USA; Department of Internal Medicine, Section of Rheumatology, Allergy & Immunology, Yale School of Medicine, New Haven, CT, USA; Department of Internal Medicine, Section of Rheumatology, Allergy & Immunology, Yale School of Medicine, New Haven, CT, USA; Department of Internal Medicine, Section of Rheumatology, Allergy & Immunology, Yale School of Medicine, New Haven, CT, USA; Department of Internal Medicine, Section of Rheumatology, Allergy & Immunology, Yale School of Medicine, New Haven, CT, USA; Department of Internal Medicine, Section of Rheumatology, Allergy & Immunology, Yale School of Medicine, New Haven, CT, USA; Department of Internal Medicine, Section of Rheumatology, Allergy & Immunology, Yale School of Medicine, New Haven, CT, USA; Department of Internal Medicine, Section of Rheumatology, Allergy & Immunology, Yale School of Medicine, New Haven, CT, USA; Department of Radiology & Biomedical Imaging, Yale School of Medicine, New Haven, CT, USA; Department of Radiology & Biomedical Imaging, Yale School of Medicine, New Haven, CT, USA; Department of Biostatistics, Yale School of Public Health, New Haven, CT, USA; Independent Statistician, Washington, DC, USA; Department of Internal Medicine, Clinical and Translational Research Accelerator, Yale School of Medicine, New Haven, CT, USA; Department of Internal Medicine, Section of Nephrology, Yale School of Medicine, New Haven, CT, USA; Department of Biomedical Informatics and Data Science, Yale School of Medicine, New Haven, CT, USA; Department of Radiology & Biomedical Imaging, Yale School of Medicine, New Haven, CT, USA; Department of Biomedical Engineering, Yale School of Medicine, New Haven, CT, USA; Department of Urology, Yale School of Medicine, New Haven, CT, USA; Yale Biomedical Imaging Institute, New Haven, CT, USA; Department of Biomedical Informatics and Data Science, Yale School of Medicine, New Haven, CT, USA; Department of Radiology & Biomedical Imaging, Yale School of Medicine, New Haven, CT, USA; Department of Biomedical Engineering, Yale School of Medicine, New Haven, CT, USA; Yale Biomedical Imaging Institute, New Haven, CT, USA; Department of Internal Medicine, Section of Rheumatology, Allergy & Immunology, Yale School of Medicine, New Haven, CT, USA; Department of Internal Medicine, Clinical and Translational Research Accelerator, Yale School of Medicine, New Haven, CT, USA; Yale Biomedical Imaging Institute, New Haven, CT, USA

**Keywords:** scleroderma and related disorders, calcified tissue, CT scanning, computer-assisted image interpretation, outcome measures

## Abstract

**Objectives:**

Calcinosis cutis (CC) is disabling for SSc patients, and quantitative outcomes and treatments are needed. We performed computer-assisted mapping of CC lesions on CT exams and quantified CC during sodium thiosulphate (STS) treatment.

**Methods:**

In a pilot study, SSc-CC patients underwent CT imaging of a painful lesion followed by 6–12 month 25% STS, intradermal monthly injections or twice daily cream, treatment. Post-treatment CT exams were obtained, and three assessors used open-source software (BioImageSuite/BIS Web) to map and quantify CC volume, termed scleroderma calcinosis cutis score (SC^2^S). Results were compared with the Scleroderma Clinical Trials Consortium Radiologic Scoring System for Hand Calcinosis as appropriate.

**Results:**

Five women with SSc-CC involving the arm, ischial tuberosities, hands (two patients) and patellae, received topical (*n* = 2) or intradermal (*n* = 3) STS. Lesion mapping with BIS Web showed high inter-rater agreement between independent assessors (intraclass correlation coefficient = 0.93). SC^2^S revealed 75% and 10% reductions in left and right buttock, respectively; 28% reduction in arm; 18% increase in left forefinger; 27% increase in right hand and 12% reduction in left, and 4% increase in right, patellae, respectively. CC volume differences on repeated measures 1-week apart were ≤3%. Mapping time ranged from <30 min for arm and buttock to >4 h for finger/hand and patellae.

**Conclusion:**

SC^2^S may be a highly reproducible, broadly applicable, quantitative outcome that is sensitive to CC change. SC^2^S highlights the variable response of SSc-CC to STS treatment. Future work to automate CC mapping will reduce lesion mapping time.

Rheumatology key messages

- Scleroderma calcinosis cutis score (SC^2^S) is a novel imaging metric to quantify calcinosis cutis volume changes.
- SC^2^S was highly reproducible among three non-expert assessors after 30 min of training.
- Calcinosis cutis change during topical or intradermal sodium thiosulphate treatment is variable.

## Introduction

Calcinosis cutis (CC) is a debilitating autoimmune disease feature particularly impacting patients with SSc. No effective SSc-CC treatment has been identified, but sodium thiosulphate (STS) is associated with improvement in lesions ≤2 cm [1]. Due to the dearth of SSc-CC quantitative outcomes, we tested a computer-aided approach for CC quantification using open-source software [BioImageSuite (BIS) Web] applied to serial CT exams during STS treatment in a pilot study [2–6]. We assessed correlations between symptoms and imaging and inter-rater and repeated measure variability.

## Methods

### Participants

Following IRB approval (HIC#2000030344), symptomatic SSc-CC patients commencing 25% STS treatment according to protocols [monthly intradermal injections following region-of-interest (ROI) anaesthesia (lidocaine 1% containing epinephrine 1:100 000) or twice daily topical cream, for 6–12 months] were enrolled (Supplementary Table S1) [7]. Pre- and post-treatment histories including CC symptom assessment and exams (e.g. painful, tender and/or draining CC), and ROI-imaging (photographs, CT, hand radiographs, as appropriate) were performed. At follow-up, ROIs were classified as improved, stable or worsened based upon physician exam. Scleroderma Clinical Trials Consortium (SCTC) Hand Radiograph Calcinosis scores were generated for patients with hand CC (a single slice of baseline CT exam was used for one lacking baseline radiography).

### BIS web software to generate SC^2^S

Digital Imaging and Communications in Medicine (DICOM) CT (www.bioimagesuiteweb.org) and radiographs were downloaded from Picture Archive and Communication System (PACS), personal identifiers removed, and exams were transferred to workstations (Supplementary Data S1). DICOM images were converted to NIfTI (Neuroimaging Informatics Technology Initiative) file format (BIS Web software requirement), using 3D Slicer (https://www.slicer.org). A *k*-means clustering algorithm in BIS Web software was applied to CT exams [each voxel (3D pixel equivalent) classified as one of three classes: 1 = air, 2 = soft tissue and 3 = bone/CC (both comprised of calcium hydroxyapatite)] [8]. Following clustering, voxel intensities of classes 2 and 3 were modelled using Gaussian distributions [by computing mean (S.D.) of each class and the intensity threshold at which the probability of belonging to each group is equal]. The BIS Web Paint Brush tool automatically applies the determined bone/CC threshold to permit its mapping.

After software tutorial, three non-expert assessors independently mapped class 3 (CC only, not bone) on a transparent image layer added atop CT images (Supplementary Methods and Fig. S1). Two radiologists (A.W. and H.L.) reviewed, and confirmed or refined, mappings.

Next, assessors generated CC ROI volume using the BIS Web Volume-of-Interest tool (termed scleroderma calcinosis cutis score [SC^2^S]). To test SC^2^S reproducibility, assessors independently generated SC^2^S on two post-treatment upper arm CT exams performed 1-week apart. Additional repetitive CT scans were avoided due to radiation risk deemed unnecessary [9, 10].

### SCTC Hand Radiographic scoring

For CC hand patients, assessors independently performed SCTC scoring [11]. The SCTC tool is a semi-quantitative method whereby raters estimate percent area involvement and average density compared with cortical bone of the middle finger middle metacarpal for 22 hand areas per hand.

### Statistical analyses

To examine SC^2^S reproducibility per time point and for repeated measures, an intraclass correlation coefficient (ICC) for raters was calculated using R version 4.4.2 (R Foundation for Statistical Computing, Vienna, Austria). The ICC calculations employed a mean-rating, absolute agreement, two-way mixed-effects model with nested random effects of side within patient SC^2^S measurements. Due to lack of detectable variability between raters, this model reduced to a one-way mixed-effects model. ICC values <0.50 were considered poor, 0.50–0.75 = moderate, 0.75–0.90 = good and >0.90 = excellent [12]. The mean (S.D.) differences between baseline and follow-up SC^2^S were generated (R Foundation for Statistical Computing, Vienna, Austria).

The minimal clinically important difference (MCID) was evaluated using the social comparison approach of the anchor-based method categorized by visual appearance change. This statistical method used the mean of two differences in CC volumetric measures, between the average ‘improved’ and ‘no change’ scores, and between the average ‘worsened’ and ‘no change’ scores [13].

### Ethics

Our study complies with the Declaration of Helsinki. Our locally appointed ethics committee has approved the research protocol (HIC#2000030344). We obtained written informed consent from the patients whose images and data are shown.

## Results

Five White biologically female patients [mean (S.D.), age = 66.6 (7.8) years with limited cutaneous SSc disease duration = 16.6 (12.4) years] were recruited (Supplementary Table S1). Mean (S.D.) STS treatment intradermal dose was 108.5 (49.6) mg. Seven CC ROI lesions were treated (two patients had bilateral lesions on hands or patellae).

### Patient-reported CC symptom and SC^2^S change over time

In three participants who reported CC symptom improvement [two treated intradermally (Cases 1 and 2) and one treated topically (case 3, left patella)], the mean (S.D.) SC^2^S decrease was 28% (31%) (Supplementary Table S2). In three patients without symptom improvement (Cases 3 (right patella), 4 and 5), the mean (S.D.) SC^2^S increase was 16% (12%). ROI density changes were concordant with SC^2^S for five out of seven lesions: a right buttock lesion treated intradermally demonstrated 10% SC^2^S improvement, but 7% density increase, while a right patella lesion demonstrated 4% SC^2^S worsening, but 4% density decrease. Of note, CT was able to accommodate resolution of small and large ROIs with voxel spacing as small as 0.293 × 0.293 × 0.6 mm^3^.

### Visual changes during treatment

**Figure 1:**
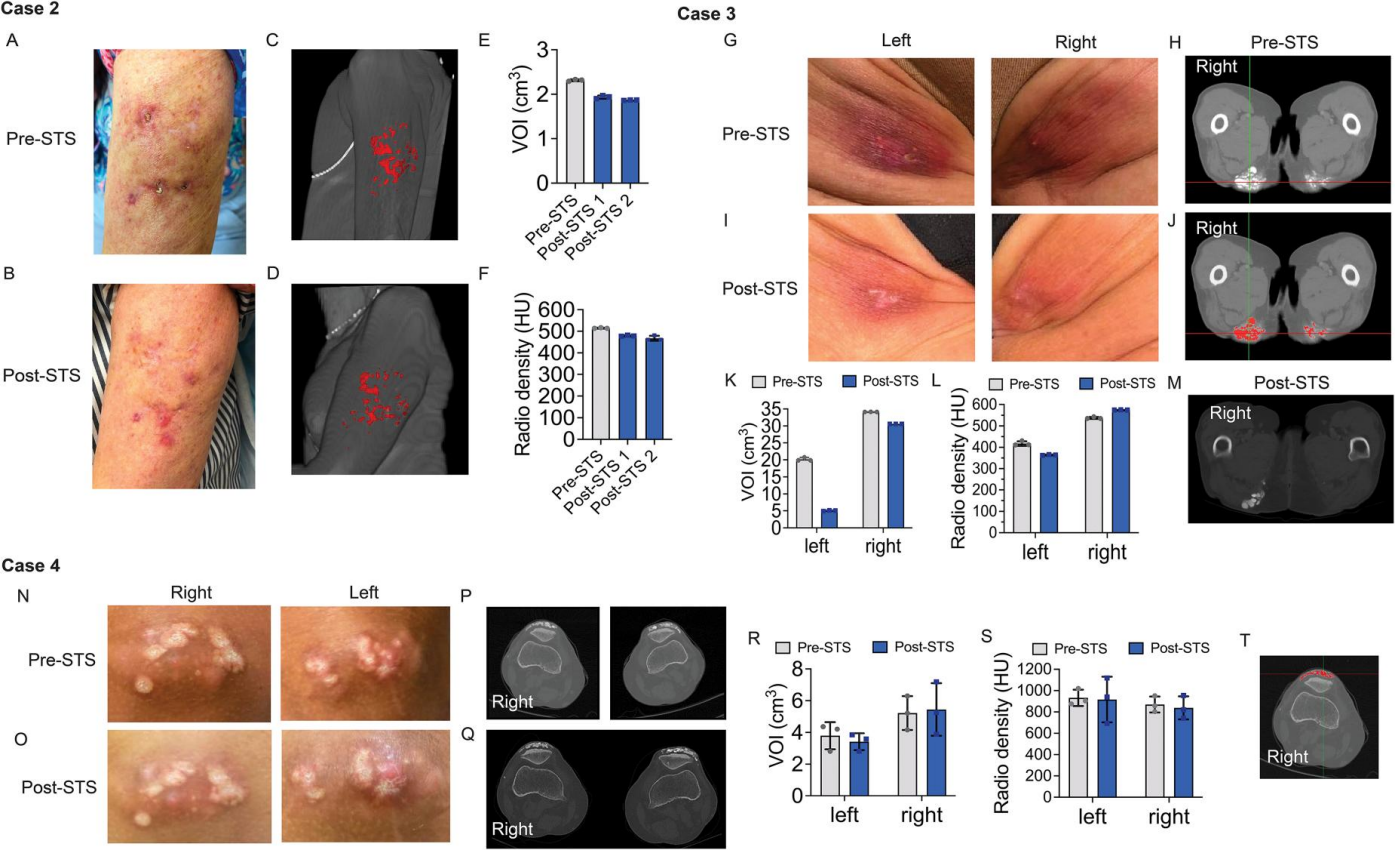
Cases demonstrating scleroderma calcinosis cutis score (SC^2^S) reduction: intradermal sodium thiosulphate (STS) treatment of upper arm (case 2) and buttocks (case 3) calcinosis cutis (CC), topical STS treatment of patellae CC (case 4). (**A, B**) Pre- and post-STS treatment photographs of left upper arm, respectively. (**C, D**) Rasterized pre- and post-STS treatment CT images of CC lesions, respectively, with CC painted red. (**E**) Pre- and post-STS treatment SC^2^S. Post-STS treatment CT exams 1 and 2 were repeated 1-week apart, VOI = volume of interest. (**F**) Average pre- and post-treatment CC lesional radio density quantification among three raters. (**G, I**) Left and right buttock photographs of pre- and post-STS treatment, respectively. (**H, J**) Pre-STS treatment axial CT images without and with painting of CC lesions. (K) Pre- and post-treatment SC^2^S, VOI = volume of interest. (**L**) Average pre- and post-treatment CC lesional radio density quantification among three raters. (**M**) Post-treatment axial CT image without painting of CC lesions. (**N, O**) Left and right patellae photographs of pre- and post-STS treatment, respectively. (**P, Q**) Pre- and post-STS treatment axial CT images of knees without painting of CC lesions. (**R**) Average pre- and post-treatment SC^2^S among three raters, VOI = volume of interest. (**S**) Average pre- and post-treatment CC lesional radio density quantification among three raters. (**T**) Pre-STS treatment axial CT image of knee with painting of CC lesion

**Figure 2:**
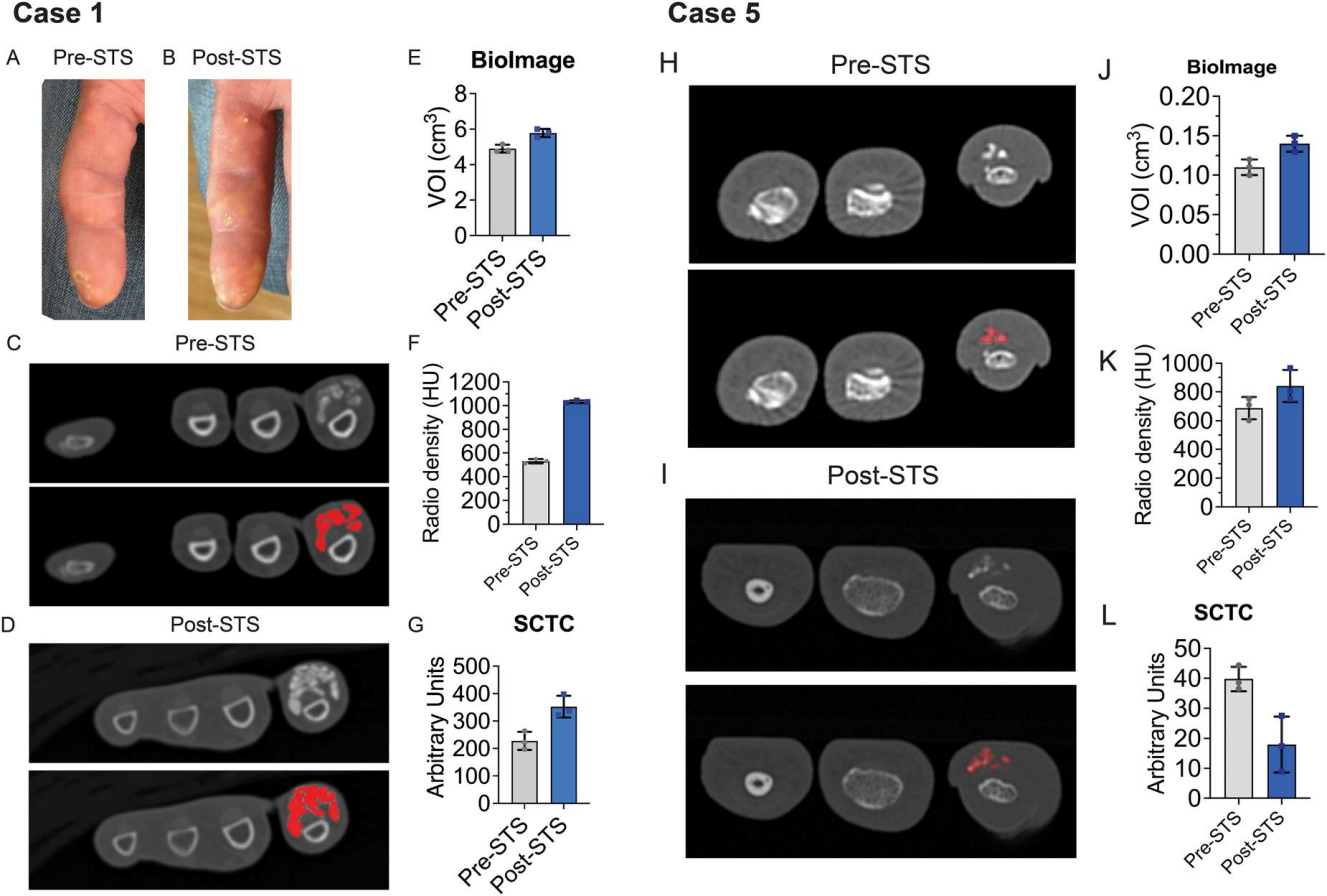
Cases demonstrating no improvement of scleroderma calcinosis cutis score (SC^2^S): sodium thiosulphate (STS) treatment of finger calcinosis cutis (CC), intradermal (case 1) and topical (case 5). (**A, B**) Pre- and post-STS treatment photographs of the right index finger, respectively. (**C**) Pre-STS treatment axial CT images without and with painting of CC lesions. (**D**) Post-treatment axial CT images without and with painting of CC lesions. (**E**) Average pre- and post-treatment SC^2^S among three raters, VOI = volume of interest. (**F**) Average pre- and post-treatment CC lesional radio density quantification among three raters. (**G**) Average pre- and post-treatment CC lesional Scleroderma Clinical Trial Consortium (SCTC) hand radiographic scores among three raters. (**H**) Pre-STS treatment axial CT images without and with painting of CC lesions. (**I**) Post-treatment axial CT images without and with painting of CC lesions. (**J**) Average pre- and post-treatment SC^2^S among three raters, VOI = volume of interest. (**K**) Average pre- and post-treatment CC lesional radio density quantification among three raters. (**L**) Average pre- and post-treatment CC lesional SCTC hand radiographic scores among three raters

Figures 1 and 2 show pre- vs post-treatment digital camera images for four of five participants (one finger CC patient lacked images) (Figs 1A, B, G, I, N, O and 2A, B). Visually, the left upper arm and bilateral buttock ROIs showed improvement, the finger and right patella ROIs lacked improvement, and the left patella ROI remained stable. Thus, SC^2^S and appearance change concordance was five out of seven (71%).

### Consistency and reliability

The SC^2^S 95% CI for two post-treatment exams was 1.85–2.02 and 1.85–1.88 cm^3^, respectively with a mean difference 0.1 cm^3^ (3% variation) (Fig. 1E). The ICC within assessors’ SC^2^S was 0.919 (95% CI: 0.739–0.966) indicating excellent reliability.

### Minimal clinically important difference

The MCID in SC^2^S over a mean (S.D.) follow-up of 9.4 (1.8) months was 25%.

### SCTC hand radiograph calcinosis scoring

Scores for the SCTC tool, applied to pre- and post-treatment studies for two participants with hand CC (three ROI) [11], showed 50% agreement with SC^2^S. One patient with right forefinger CC showed 55% SCTC score increase that mirrored 18% SC^2^C increase (Supplementary Table S2). However, the bilateral finger CC patient showed 55% SCTC score decrease but 27% SC^2^S increase.

## Discussion

Patients with SSc-CC receive unproven therapies resulting in exposure to ineffective and/or harmful treatment. Currently, physicians rely on clinical assessments, patient-report of symptoms, hand radiographs, digital camera imaging and/or more advanced imaging methods including CT to estimate change. While patient-report of symptom change is the gold-standard treatment assessment, quantitative outcomes are needed for clinical trials. We conducted a pilot study in five patients with symptomatic SSc-CC and tested the ability of SC^2^S, a novel tool for CC quantification developed at our institution, to measure CC change in different body locations. We tested the association between symptom and imaging changes, assessed consistency and reliability, and explored the minimal clinically important difference. Our results suggest that SC^2^S may be feasible, reproducible and sensitive to change, but our numbers are small, and our results require validation in larger studies.

The dearth of quantitative outcomes is a key barrier to SSc-CC clinical trials. To date, no quantitative methods to assess CC burden have been developed and validated. The Mawdsley Calcinosis Questionnaire (MCQ), under development since 2015 with FDA guidance, was based upon qualitative interviews of 31 patients living with SSc-calcinosis [14, 15]. The final questionnaire includes 19 questions for CC quantity/frequency, pain/sensation, physical function and psychological impact [14, 15]. Although potentially useful as an outcome for a CC systemic therapy, local, rather than systemic, treatment in our study precluded MCQ use.

For two patients with hand CC, we employed the SCTC tool to estimate CC change over time. Strengths of the SCTC tool include, (1) cost effectiveness, (2) lower ionizing radiation exposure compared with CT, (3) speed and ease of CC estimate generation, (4) demonstrated sensitivity to hand CC change over 1 year [11] and (5) its validation [11, 16]. Weaknesses of the SCTC tool include its restricted use in hand radiographs, semi-quantitative nature and inability to quantify changes in volume that may precede density and/or area estimated change. Underscoring this point, only one of two patients with hand CC had concordant SC^2^S and SCTC score changes. In addition to greater sensitivity, BIS Web software can be used to quantify CC density. The importance of measuring CC volume and density is underscored by case 2 where volume reduction of the right buttock CC lesion coincided with increased density suggesting STS treatment was less efficacious than volume measurements alone would suggest.

CT exams were performed for this study as opposed to MRI because CT is more accessible, less expensive and not confounded by sequence variance. CT was preferred over US because US is operator dependent, and thus image acquisition is not standardized (as opposed to CT images that are acquired from calibrated scanners), and the field-of view is small which complicates assessment of large CC ROIs (e.g. arms and buttocks).

We used interactive image editing tools in BIS Web software (successfully employed for many indications [2–6]) to manually map CC on CT exams. BIS Web software leverages the 3D nature of CT permitting users to view and paint image voxels containing CC (mapping) on an axial plane image for instance while simultaneously viewing annotations on coronal and sagittal planes. This feature vastly simplifies the problem of differentiating CC from bone that is particularly important in areas where CC is adjacent to bone (e.g. hands and knees). Moreover, because BIS Web tools are 3D, CC mapping performed on one slice appears on subsequent slices requiring users only to make fine adjustments (e.g. painting/unpainting a voxel on sequential CT image slices). Additionally, BIS Web permits users to paint desired areas with different sized paint brushes to accommodate large and small CC lesions. Once CC is mapped, the software rapidly generates volume-of-interest (SC^2^S) and mean density-of-interest.

Mapping of CC on CT imaging of arm and buttock lesions was simple and efficient to perform due to low radiographic density of tissue between arm CC and the humerus, and buttock CC and ischial tuberosities, respectively. Conversely, mapping of hand and knee CC was more challenging and time-consuming because of the proximity and similar radiodensity of finger CC lesions to phalanges, and knee CC lesions to patellae. To address this pitfall, we are developing semi-automated approaches to facilitate phalangeal segmentation that will greatly reduce the time for SC^2^S of the hands, the most commonly involved body region [17]. This may also have potential to be translated to other lesions close to bone besides the hand.

We showed variable CC improvement during STS treatment that echoes our clinical experience. Our results support, but do not confirm, the test–retest and inter-rater reliability of SC^2^S using BIS Web tools. The test–retest reliability is supported by the strong ICC and narrow 95% CI for the two post-treatment arm exams, although we only had repeated CT measures for one patient. The risk of additional radiation exposure will have to be weighed against the need for additional test–retest validation. Ongoing studies include multiple raters to further assess intra- and inter-rater SC^2^S reproducibility.

In this pilot study of five patients, validated patient- and/or physician-reported outcome measures were lacking [15], however, a larger funded study is currently underway (please see Clinical Trials.gov, NCT07228429, for additional information). Discordance between patient-reported symptom improvement and CC volumetric change also warrants additional investigation into how factors such as CC structure and density, and interactions with the surrounding dermis, may contribute to symptoms. Study strengths include use of standardized STS topical and intradermal treatment protocols, CC mapping by three independent assessors blinded to pre- or post-treatment CT exam designation, and confirmation of accurate CC mapping by two expert musculoskeletal radiologists. An additional strength is comparison between the SC^2^S and the SCTC tool for both patients with hand CC.

In conclusion, we present results that demonstrate that SC^2^S, a novel CC outcome, may be useful for quantifying CC change in treated SSc patients. Results from our larger ongoing study will report additional SC^2^S performance characteristics that may pave the way towards SSc-CC randomized controlled clinical trials.

## Supplementary Material

keag302_Supplementary_Data

## Data Availability

Imaging and patient level data can be made available upon reasonable request to the corresponding author.

## Data access, responsibility and analysis

I.O. and M.H. had full access to all the data in the study and take responsibility for the integrity of the data and the accuracy of the data analyses.
