# Supplementary material for: Scleroderma calcinosis cutis score (SC2S): an imaging metric to quantify SSc-calcinosis cutis
Source: Rheumatology (Oxford). 2026 Jun 11;65(6):keag302. doi: 10.1093/rheumatology/keag302 (PMC13290485; doi:10.1093/rheumatology/keag302)

**Supplementary Materials:**

**Supplementary Data S1: Methods**

Protocol: Calcinosis Cutis Painting/mapping/annotating using BIS Web Software

1. Digital Imaging and Communications in Medicine (DICOM) de-identified CT images are exported from Picture Archive and Communication System (PACS)
2. Convert DICOM CT images to NIfTI format (.nii or .nii.gz)
   1. This can be done in the 3D Slicer open-source program (https://www.slicer.org/), but any program/code that can do this also works
3. Open Image Editor application in BIS Web (<https://bioimagesuiteweb.github.io/webapp/editor.html>)
4. Load NifTI format image under File

**Example:**

**
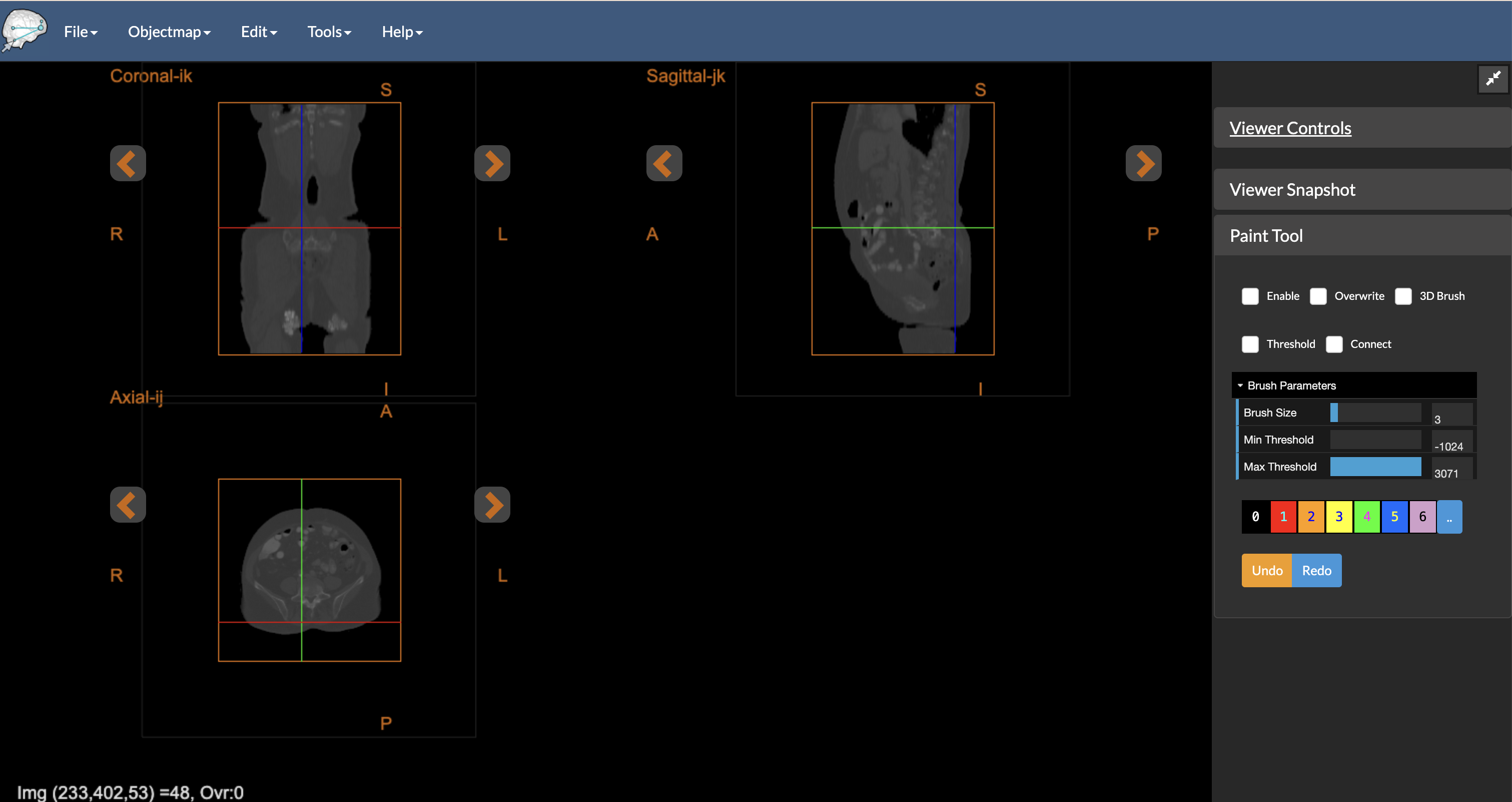
**

1. Under Paint tools:
   1. Enable painting
   2. Select 1 (red)
   3. Select “Threshold”, set minimum threshold to 100
      1. Minimum threshold constrains brush to only paint intensities above the value selected
   4. Select “3D Brush”
      1. 3D Brush extends brush to multiple slices based on brush size (*e.g.,* brush size 3 will paint 3x3x3 in 3D)

**Example:**

**
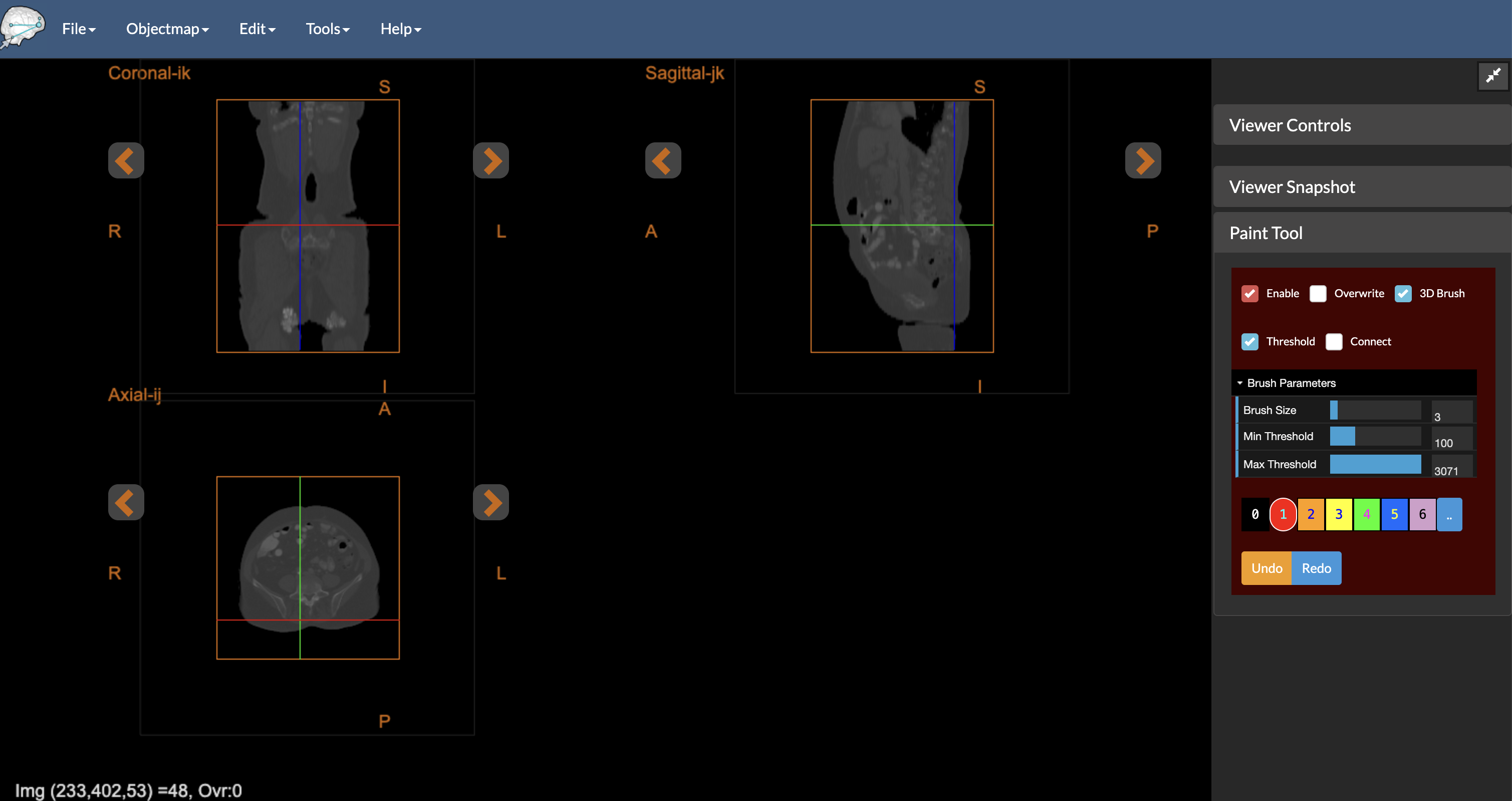
**

1. Under Viewer Controls tool, set opacity in overlay color mapping to 0.3
   1. This allows the user to see the calcinosis lesion as you paint

**Example:**

**
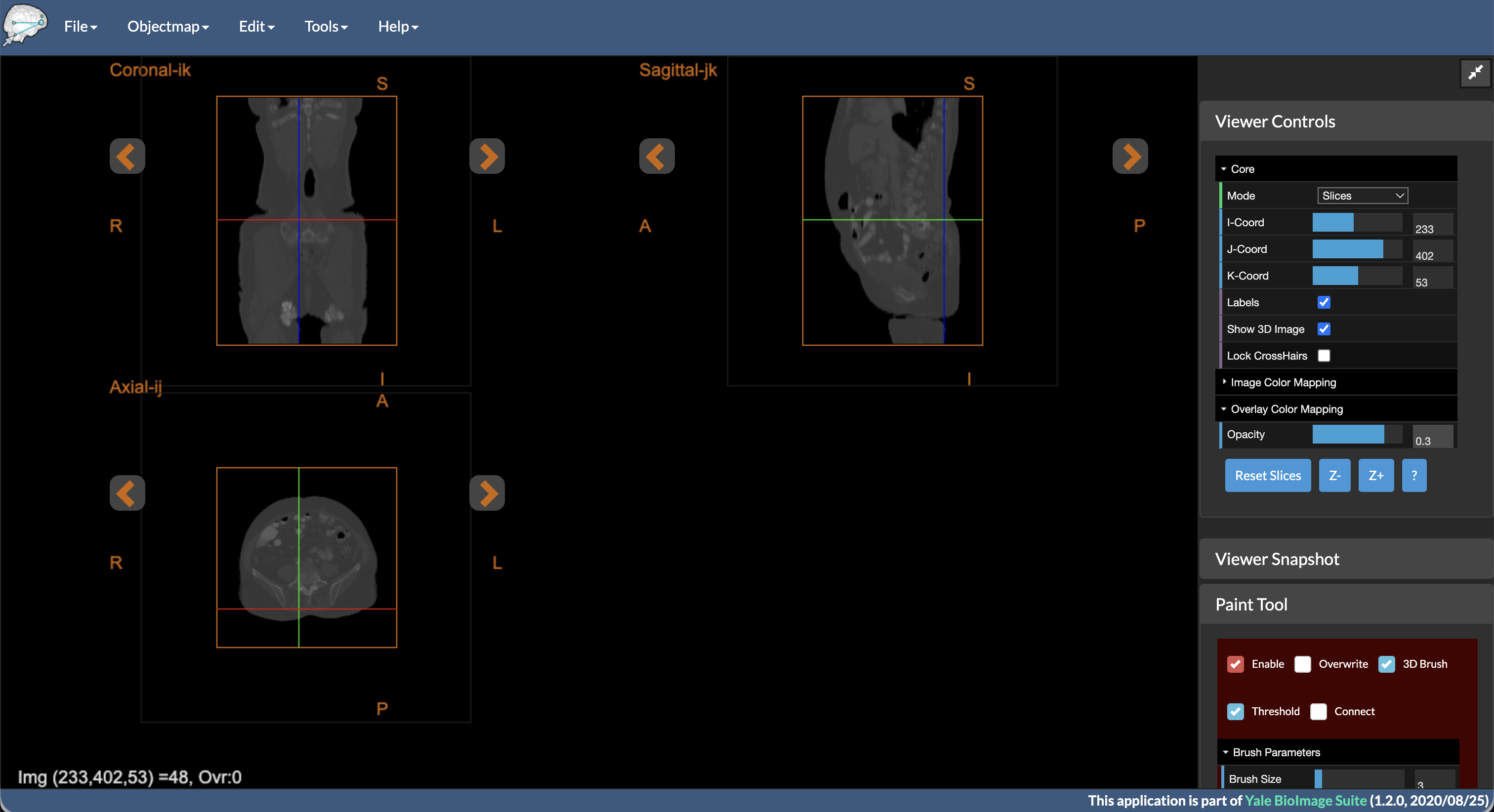
**

1. Use mouse to paint on CT slice
   1. Set brush to 3 pixels
   2. Moving slices: Z- or Z+ can change slices or the left/right arrows
   3. Moving image position: Dragging with two fingers on trackpad (right click on a mouse) can move the CT image around
   4. Zooming in: Spread two fingers apart on trackpad with the mouse over the CT image (scroller on a mouse)
   5. Click and hold on image area with calcinosis to paint
   6. Go through each slice to paint
   7. Set brush to 1 pixel to paint edges missed

**Example:**

**
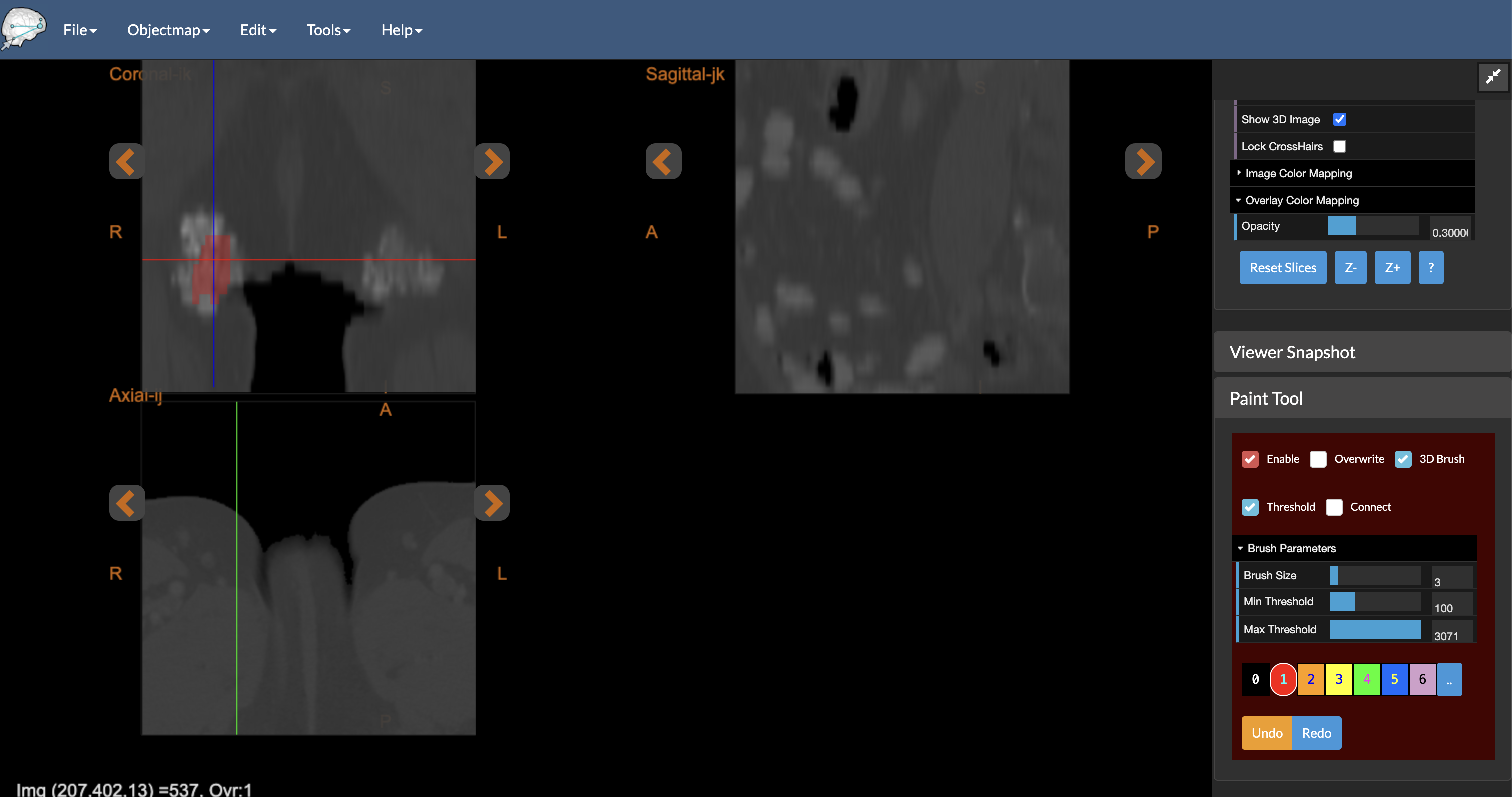
**

1. For overpainted edges, in paint tool:
   1. Set brush to 1 pixel
   2. Enable overwrite
   3. Select 0 (black)
   4. Paint over the red pixels you want to erase
   5. Deselect overwrite to go back to painting in red
2. Volume/density analysis
   1. Under Objectmap, select “VOI Analysis”
   2. The first graph that shows up is the average radiodensity

**Example:**

**
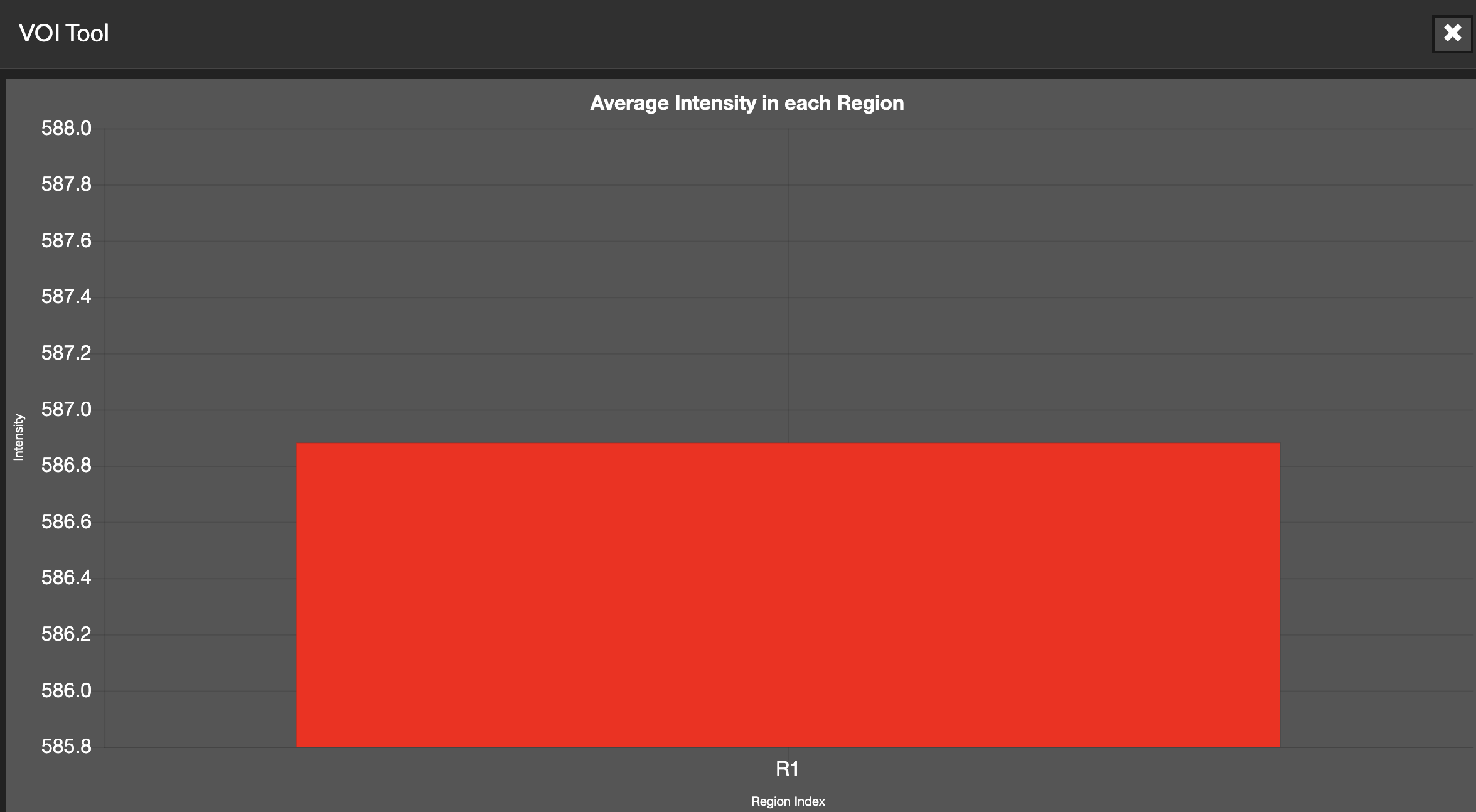
**

- 1. Select “Plot VOI volumes” in the bottom right, for the volume quantification

**Example:**

**
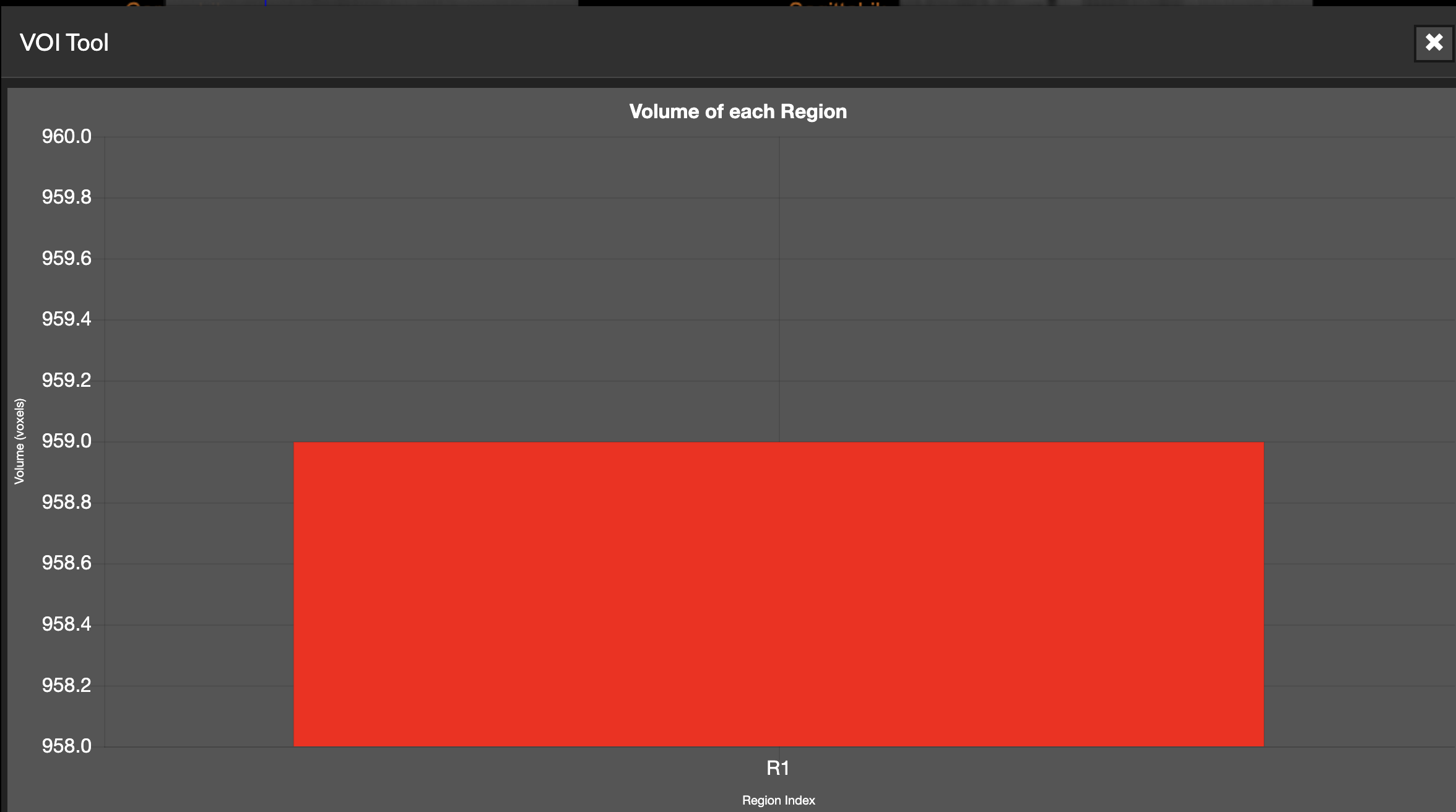
**

1. Saving files
   1. Under Objectmap, click “save Objectmap”
      1. This saves the painting layer as a NifTI file
   2. Next time the user wants to open the painting, load the image then load the objectmap
      1. This loads the original base CT image and then loads the painting layer atop it

**Supplementary Table S1**

| **Supplementary Table 1: Demographics** | |
| --- | --- |
| **N (%) or as indicated** | **Overall (n=5)** |
| **Age, years, mean (SD)** | 66.6 (7.80) |
| **Women** | 5/5 (100%) |
| **White** | 5/5 (100%) |
| **Limited cutaneous SSc** | 5/5 (100%) |
| **SSc disease duration, years, mean (SD)** | 16.6 (12.4) |
| **ANA positive**   - **Centromere pattern** - **Nucleolar pattern** - **Speckled pattern** | 2/5 (40%)  1/5 (20%)  1/5 (20%) |
| **Anti-Scl70 positive** | 0/5 (0%) |
| **Anti-RNA polymerase III** | 0/5 (0%) |
| **ACA positive** | 2/5 (40%) |
| SD (standard deviation), ANA (antinuclear antibody), ACA (anticentromere antibody). One participant had no antibody testing on file. | |

**Supplementary Table S2**

| **Supplementary Table 2: Imaging and symptom changes during sodium thiosulfate treatment** | | | | | | | | | | | | |  |
| --- | --- | --- | --- | --- | --- | --- | --- | --- | --- | --- | --- | --- | --- |
| **Participant** | **CC lesion of interest** | **Visual appearance change** | **Patient-reported symptom change** | **CT volume quantification** | | | **CT radiodensity** | | | **SCTC hand radiograph** | | |  |
|  |  |  |  | **Pre, cm^3^, mean (SD)** | **Post, cm^3^, mean (SD)** | **SC²S (%)** | **Pre, HU, mean (SD)** | **Post, HU, mean (SD)** | **% change** | **Pre, AU, mean (SD)** | **Post, AU, mean (SD)** | **% change** |  |
| **One (I)** | L upper arm | improved | improved | 2.31 (0.2) | 1.93 (0.4) | -16% | 514 (2) | 479 (3) | -7% | -- | -- | -- |  |
|  |  |  |  | -- | 1.87 (0.01)^a^ | -- | -- | -- | -- | -- | -- | -- |  |
| **Two (I)** | L buttock | improved | improved | 20.1 (0.51) | 5.02 (0.12) | -75% | 418 (11) | 364 (2) | -13% | -- | -- | -- |  |
|  | R buttock | improved | improved | 34.1 (0.01) | 30.6 (0) | -10% | 538 (5) | 574 (1) | +7% | -- | -- | -- |  |
| **Three (T)** | L patella | no change | improved | 3.8 (0.85) | 3.4 (0.53) | -11% | 932 (76) | 916 (214) | -2% | -- | -- | -- |  |
|  | R patella | worsened | no change | 5.2 (1.07) | 5.4 (1.65) | +4% | 869 (75) | 838 (108) | -4% | -- | -- | -- |  |
| **Four (I)** | R second finger | worsened | worsened | 4.9 (0.22) | 5.8 (0.23) | +18% | 530 (18) | 1032 (11) | +95% | 227.8 (33.2) | 352.8 (39.5) | +55% |  |
| **Five (T)** | B/l fingers | worsenedᵇ | worsened | 0.11 (0.01) | 0.14 (0.01) | +27% | 688 (77) | 841 (112) | +22% | 39.8 (4.1) | 17.9 (9.3) | -55% |  |
| CC (calcinosis cutis), SD (standard deviation), SC²S (scleroderma calcinosis cutis score), (HU (Hounsfield units), SCTC (Scleroderma Clinical Trials Consortium), (AU (arbitrary units), I (intralesional STS), T (topical STS), R (right), L (left), B/l (bilateral).  ^a^ Repeat CT taken one week after first post-CT for participant one.  ᵇ No photo available, physician-reported visual change. | | | | | | | | | | | | |  |
|  |  |  |  |  |  |  |  |  |  |  |  |  |  |
|  |  |  |  |  |  |  |  |  |  |  |  |  |  |

**Supplementary Figure S1. Coronal, sagittal, and axial views of patellae CC case in BIS Web software with CC lesion painted in red.**


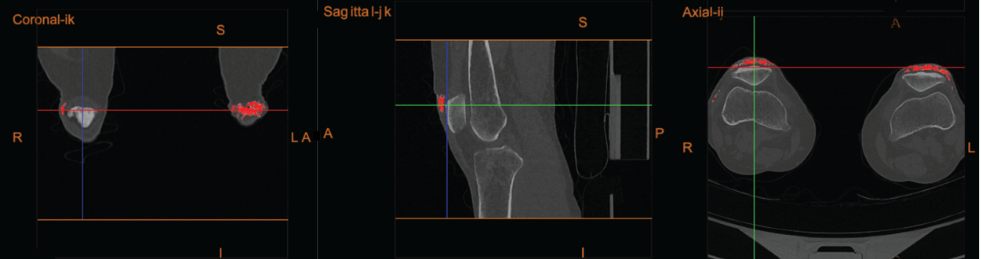

Supplement: keag302_Supplementary_Data [file keag302_supplementary_data.docx]
